# Signaling crosstalk between tumor endothelial cells and immune cells in the microenvironment of solid tumors

**DOI:** 10.3389/fcell.2024.1387198

**Published:** 2024-04-25

**Authors:** Yuexin Xu, Chris P. Miller, Scott S. Tykodi, Shreeram Akilesh, Edus H. Warren

**Affiliations:** ^1^ Translational Science and Therapeutics Division, Fred Hutchinson Cancer Center, Seattle, WA, United States; ^2^ Department of Medicine, Division of Hematology and Oncology, University of Washington, Seattle, WA, United States; ^3^ Clinical Research Division, Fred Hutchinson Cancer Center, Seattle, WA, United States; ^4^ Department of Laboratory Medicine and Pathology, University of Washington, Seattle, WA, United States; ^5^ Kidney Research Institute, University of Washington, Seattle, WA, United States

**Keywords:** tumor endothelial cell, angiogenesis, immune cell transmigration, immune suppression, tumor microenvironment across the tumor endothelium

## Abstract

Tumor-associated endothelial cells (TECs) are crucial mediators of immune surveillance and immune escape in the tumor microenvironment (TME). TECs driven by angiogenic growth factors form an abnormal vasculature which deploys molecular machinery to selectively promote the function and recruitment of immunosuppressive cells while simultaneously blocking the entry and function of anti-tumor immune cells. TECs also utilize a similar set of signaling regulators to promote the metastasis of tumor cells. Meanwhile, the tumor-infiltrating immune cells further induce the TEC anergy by secreting pro-angiogenic factors and prevents further immune cell penetration into the TME. Understanding the complex interactions between TECs and immune cells will be needed to successfully treat cancer patients with combined therapy to achieve vasculature normalization while augmenting antitumor immunity. In this review, we will discuss what is known about the signaling crosstalk between TECs and tumor-infiltrating immune cells to reveal insights and strategies for therapeutic targeting.

## Introduction

**FIGURE 1:**
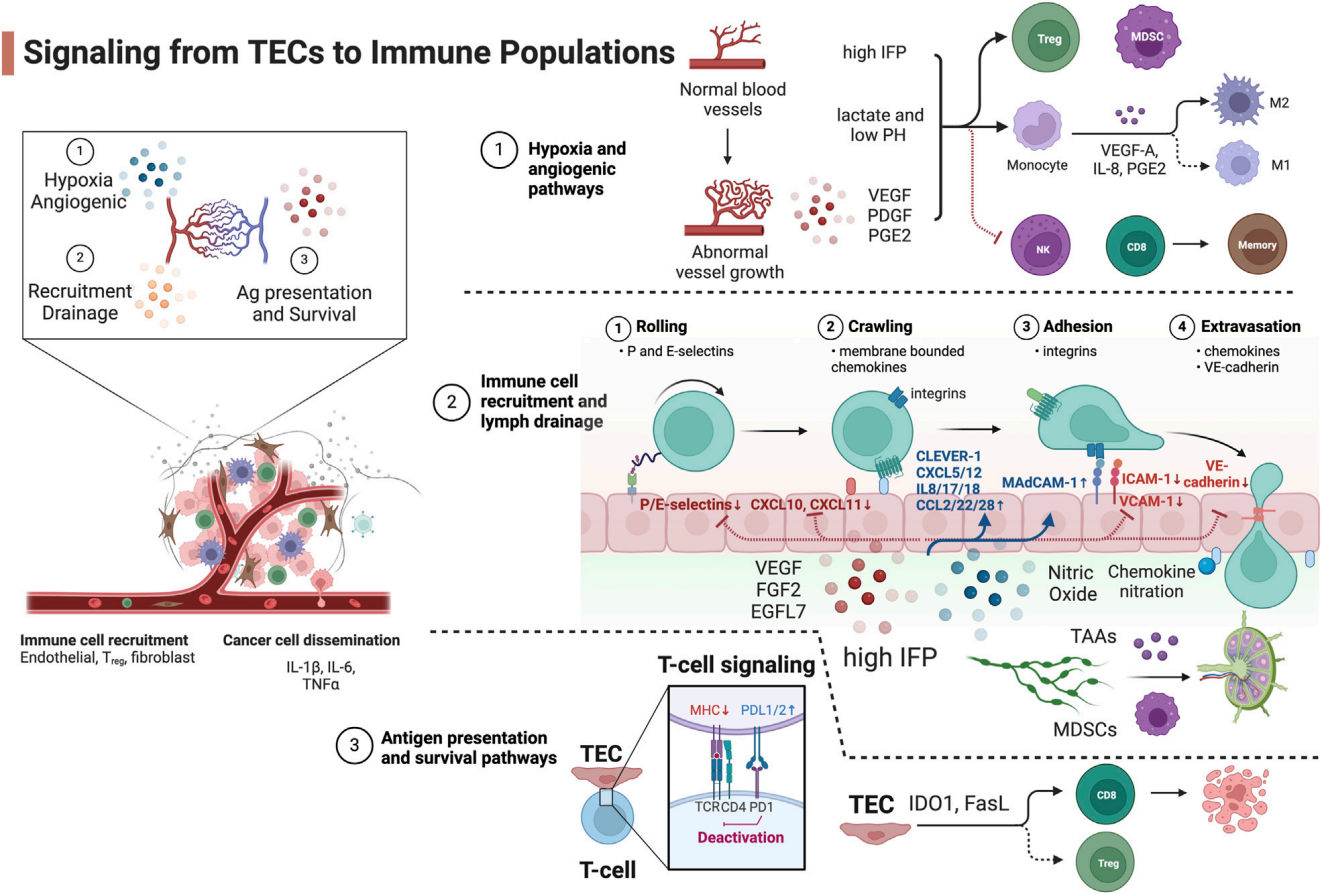
Signaling from TECs to immune populations. Blue, upregulated signaling molecules in TECs. Red, attenuated signaling molecules in TECs. Created with BioRender.com.

The endothelial cell network represents the first barrier for circulating immune cells to enter the tissue microenvironment through the process of transendothelial extravasation and migration under inflammatory conditions (Ley et al., 2007). It is well established that tumor-associated endothelial cells develop anergic phenotypes (Griffioen et al., 1996) that upregulate neo-angiogenesis, extracellular matrix (ECM) degradation, and IGF regulation pathways (Xu et al., 2023) while down-regulating adhesion molecules and interferon signaling pathways to selectively limit certain type of immune cell entry, which in turn profoundly impacts the immune cell composition within the TME (Huinen et al., 2021). The T cell populations crossing the endothelial barrier can be selected by TECs via specialized chemokines and chemokine receptors. Contact with TECs can also regulate T cell differentiational status and functional capacity. Tumor-associated macrophage (TAM) infiltration positively correlates with blood vessel density in the TME (Yang et al., 2021). In contrast, the presence of an adaptive anti-tumor immune response negatively impacts the function and the angiogenic phenotype of TECs. The emergence of combined therapies targeting angiogenesis and augmented T cell immunity with immune checkpoint blocking antibodies highlights the importance of understanding the crosstalk between the endothelial system and immune components when administrating therapy to cancer patients (Yi et al., 2019). The normalized tumor vascular network promotes T cell recruitment and induces M1-like TAM polarization (Huang et al., 2012). In this mini review, we will discuss the signaling crosstalk between the TECs and immune components in the TME. The molecular interactions between TECs and immune cells serve as a rich landscape of targets for anti-tumor therapeutic development (Figure 1, created with BioRender.com).

## Signaling from TECs to immune cell subsets

### Hypoxia and angiogenic pathways

Rapid oxygen consumption by fast-growing tumor cells stimulates pro-angiogenic signaling pathways. The hypoxia in tumor regions that are farthest from the tumor vasculature initiates proangiogenic gene expression such as platelet-derived growth factors (PDGFs) (Kourembanas et al., 1990) and vascular endothelial growth factors (VEGFs) (Shweiki et al., 1992) via the hypoxia-inducible factor (HIF) transcription factors (Pugh and Ratcliffe, 2003). These signaling molecules stimulate the angiogenic vasculature reprogramming via VEGF or the angiopoietin (ANG1/2)/Tie-2 pathway (Augustin et al., 2009) in an autocrine or paracrine manner. Angiogenesis is one of the hallmarks of cancer and is associated with tumor vessels that are abnormal. There are direct interactions of pro-angiogenic molecules with immune cell populations to foster an immune privileged microenvironment. The circulating HIF-1 target VEGF-A impairs the maturation and function of DCs which impedes antigen-presentation (Gabrilovich et al., 1996) mediated by the inhibition of nuclear factor kappa-light-chain-enhancer of activated B cells (NF-κB) (Oyama et al., 1998). In response to the high VEGF-A level, myeloid-derived suppressor cells (MDSCs) expand and acquire immunosuppressive features (Kusmartsev et al., 2008). The upregulated VEGF-A and prostaglandin E2 (PGE2) expression also stimulate the TECs to secrete IL-8 which drives TAM M2-polarization (Martinez et al., 2008). Moreover, the pro-angiogenic PDGF-C enhances TAM survival by inhibiting the activation of caspase-3, -7, -8, and -9 and cleavage of poly (ADP-ribose) polymerase (Son et al., 2014).

The angiogenic vasculature in tumor is often leaky, collapsed with narrowed lumens, and ill-perfused with blunt ends and low blood flow (Schaaf et al., 2018). These abnormal vessels elevate the interstitial fluid pressure (IFP) that increases transforming growth factor beta (TGF-β) production from local fibroblasts (Swartz and Lund, 2012) or myeloid dendritic cells (DCs) which maintains an immature DC phenotype, stimulates proliferation of regulatory T cell (Tregs) (Ghiringhelli et al., 2005), and induces tumor-associated macrophage (TAM) M2-phenotype reprogramming via interleukin one receptor-associated kinase (IRAK)-M expression (Standiford et al., 2011).

The TECs are inefficient at delivering nutrients, exchanging oxygen (Arner and Rathmell, 2023) and removing waste. This in turn exacerbates the deprivation of local oxygen and causes a profound switch of metabolic pathways from oxidative phosphorylation to anaerobic glycolysis via mammalian target of rapamycin complex one signaling dysregulation and contributes to an immunosuppressive microenvironment. The inability to remove metabolic waste results in the accumulation of lactate and decreases the pH of the extracellular milieu. An acidic TME inhibits the differentiation of CD8^+^ T cells to memory T cells (Brand et al., 2016) and the function of natural killer (NK) cells (Gerriets et al., 2016), promotes the survival of Tregs (Colegio et al., 2014) and polarizes TAMs toward an immunosuppressive M2-like phenotype (Nakagawa et al., 2015).

### Immune cell recruitment and lymph drainage pathways

The endothelial barrier controls tumor cells dissemination and immune cell infiltration. TECs have aberrant expression of key adhesion molecules that favors tumor cell intravasation and suppresses immune cell extravasation. The process of immune cell transmigration consists of the following steps: rolling (mediated by P/E-selectin), intravascular luminal activation and crawling (mediated via membrane bound chemokine - chemokine receptor interaction), adhesion (mediated via integrin - VCAM-1/ICAM-1 interaction), and paracellular or intracellular extravasation (Salminen et al., 2020).

Angiogenic TECs develop an altered phenotype that prevents immune cell infiltration into the TME. VEGF-A (Flati et al., 2006), fibroblast growth factor 2 (FGF2) (Flati et al., 2006), and epidermal growth factor-like domain-containing protein 7 (EGFL7) (Delfortrie et al., 2011) actively downregulate the expression of the leukocyte adhesion molecules P/E-selectin, ICAM1 and VCAM1 on the luminal side of TECs (Griffioen et al., 1996). This results in TECs that are unable to tether leukocytes, and eventually hampers leukocyte infiltration. Another signaling molecule negatively regulating immune cell recruitment and drainage is nitric oxide (NO) secreted by TECs, MDSCs, and TAMs. NO is a potent vessel relaxant that reduces blood flow. High levels of NO significantly affect leukocyte recruitment by downregulating ICAM-1 expression, preventing immune cell rolling and adhesion (De Caterina et al., 1995). The overexpression of endothelin B receptor and the ligand endothelin-1 in tumors increases NO release in the endothelium, thereby impairing lymphocyte arrest and reducing tumor-infiltrating lymphocyte content in the tumor (Buckanovich et al., 2008). Notably, NO can also attenuate the contraction cycles of tumor-associated lymphatic vessels (Liao et al., 2011), reducing tumor antigen-presenting DC drainage and impairing T-cell priming in the draining lymph node. The low lymphatic drainage in turn elevates the IFP that exacerbates the immunosuppressive microenvironment (Raju et al., 2008) by the mechanism mentioned above. Another body of evidence demonstrates that high IFP increases lymph drainage to promote the recruitment of tumor-associated antigens and immature DCs from the immunosuppressive microenvironment to the adjacent lymph nodes and activates naïve T cells in a tolerogenic manner to compound peripheral tolerance (Tammela and Alitalo, 2010; Swartz and Lund, 2012). The two mechanisms may both function in solid tumors, and spatial-temporal regulation of lymphatic drainage in different tumor types and disease stages needs to be further investigated. On the contrary, TECs selectively upregulate specific adhesion molecules that promote infiltration of immunosuppressive cells. TECs upregulate mucosal addressin cell adhesion molecule 1 (MAdCAM1), which interacts with β7 integrin expressed on Tregs, thereby preferentially promoting Treg infiltration into tumor sites (Nummer et al., 2007). An example is tumor-associated lymphatic endothelium upregulation of immunosuppressive adhesion receptor common lymphatic endothelial and vascular endothelial receptor (CLEVER-1/STABILIN-1), resulting in the infiltration of Tregs (Nummer et al., 2007) and TAMs (Karikoski et al., 2014).

Chemokine and chemokine receptor signaling-mediated intravascular crawling is well recognized as the key selection step for the preferential recruitment of specific immune populations. The levels of CXCL10, CXCL9, and CXCL11 in the tumor correlates with the concentration of intratumoral cytotoxic lymphocytes (CTLs) and reduced tumor angiogenesis (Burkholder et al., 2014). TECs upregulate chemokines (CXCL12 and sphingosine one phosphate) and receptors (CXCR4/ACKR3/CXCR2 and sphingosine one phosphate receptor) via HIF-1 signaling to drive the blood vessel branching and chronic activation of the endothelium in response to the initial vasculature reprogramming factors (Ceradini et al., 2004; Potente et al., 2011). These proangiogenic chemokine and chemokine receptors promote tumor cell dissemination (Guo et al., 2016) and regulate the entry and function of immunosuppressive cells. CXCL12 recruits regulatory B cells into the tumor which can exacerbate tumor progression via IL-10 and TGF-β expression (Qin et al., 2015). These recruited regulatory B cells also induce CXCR4 expression on tumor cell to promote cancer cell metastasis to the lymph node by increase the responsiveness to the pro-metastatic chemokine CXCL12 (Gu et al., 2019). Moreover, CXCL12 released by TECs and BoxA, a fragment of HMGB1, can engage the CXCR4-CD47 complex and trigger CD47 internalization to release a phagocytosis signal by the tumor cells and attract macrophages (Mezzapelle et al., 2021). While this machinery may elicit antitumor immunization via antigen presentation on macrophages, the phenotype polarization of the CXCL12/CXCR4 recruited TAMs can be immunosuppressive and needs to be further characterized.

To selectively recruit immunosuppressive cells, TECs secrete IL-8 (CXCL8, receptor CXCR1/2) to induce EC proliferation in an autocrine manner, which also results in disrupted EC intercellular junctions (Dwyer et al., 2012). The high level of IL-8 activates β2 integrins (CD18) to arrest neutrophils during transmigration (DiVietro et al., 2001), attracts immunosuppressive CXCR2+ N2-phenotype tumor-associated neutrophils (Alfaro et al., 2016), and stimulates the formation of neutrophil extracellular traps. It also preferentially recruits MDSCs and promotes M2-phenotype polarization (Ostrand-Rosenberg and Fenselau, 2018). Aside from IL-8, additional factors including IL-1β, CCL2, CXCL5, IL-17 and IL-18 also selectively recruit subsets of MDSCs from the vasculature into renal cell carcinoma (Najjar et al., 2017; Guan et al., 2018) while CCL3 and CCL5 are important for the retention of MDSCs in tumors (Kumar et al., 2016). The soluble pro-angiogenic molecule VEGF-A also acts as an atypical chemoattractant to facilitate MDSC recruitment (Wang et al., 2019). The hypoxic TME also induces the upregulation of CCL22 and CCL28 from TECs, which preferentially recruit Tregs into solid tumors (Curiel et al., 2004; Facciabene et al., 2011). Moreover, semaphorin 3A is another non-canonical attractant induced by hypoxia and promotes the retention of TAMs in lung cancer (Casazza et al., 2013). On the opposite side, VEGF-A suppresses pro-inflammatory T-cell infiltration into the tumor through inhibition of NF-κB and TNF-α-induced downregulation of CXCL10 and CXCL11 (Huang et al., 2015). Moreover, chemokines secreted by TECs can be post-translationally modified to preferentially recruit immunosuppressive cells. The nitration of CCL2 in tumors can suppress T cell infiltration, while macrophages and MDSCs can still be recruited by nitrated CCL2 (Molon et al., 2011).

On the step of extravasation, the tumor vasculature has developed strategies to break the balance of tumor cell metastasis and leukocyte infiltration. The dysfunctional and leaky tumor vasculature that promotes fluid extravasation, high IFP, and eventual tumor metastasis also inversely correlates with the accumulation of tumor-infiltrating lymphocytes (TILs) in the TME (Park et al., 2016). VE-cadherin (cadherin-5, CD144) is an adherence junction protein ensuring proper barrier function and primarily mediates the paracellular route of leukocyte transmigration (Gavard, 2013) and tumor cell metastasis by forming cell-cell junction gaps. TECs downregulate the expression of VE-cadherin resulting in a disrupted endothelial barrier with reduced functional CD8 T cell infiltration routes into tumor (Zhao et al., 2017). Yet studies have demonstrated that metastatic tumor cells can induce local gap formation by activating Src to subsequently phosphorylate VE-cadherin (Potter et al., 2005; Aragon-Sanabria et al., 2017), providing a route for tumor cells to pass through the endothelial cell layer into the vessel lumen.

### Antigen presentation and survival pathways

TECs are known to regulate T cell activation pathways by altering the expression of antigen-presentation complexes or inhibitory molecules that deactivate T cell function when entering the TME. T cell receptor signaling is the key pathway to elicit an anti-tumor T cell response. Although ECs are atypical antigen-presenting cells: they constitutively express major histocompatibility complex (MHC) class I and II but in general have limited co-stimulatory CD80 and CD86 expression which are required for naïve T cell activation (Denton et al., 1999; Xu et al., 2024). It has been reported that the TECs downregulate MHC I and II molecules which negatively impacts T cell priming (Lambrechts et al., 2018). Importantly, the tumor vasculature and angiogenic factors modulate the activated T cell functions by expressing a wide range of co-inhibitory and co-stimulatory molecules that foster anergy in the TIL. While the expression of cell-surface programmed death ligand one and 2 (PD-L1 and PD-L2) on tumor blood and lymphatic ECs controls vessel damage from the activated and extravasating T cells (Rodig et al., 2003), they also contribute to TIL anergy and foster an immunosuppressive microenvironment. VEGF-A can upregulate the expression of PD-1, TIM-3, and CTLA-4 on tumor-infiltrating CD8^+^ T cells (Voron et al., 2015) via the VEGFR2-PLCγ-calcineurin-NFAT pathway. This machinery explains the synergistic effect of tumor growth suppression when administering anti-PD-1 therapy together with anti-VEGF-A. CD137 (4-1BB) is a TCR signaling co-stimulatory molecule expressed by ECs that induces adhesion molecule expression. Tumor cells enhance the expression of soluble CD137 that competes with the membrane bound CD137 as an antagonist of T cell co-stimulation and activation (Hentschel et al., 2006).

**FIGURE 2:**
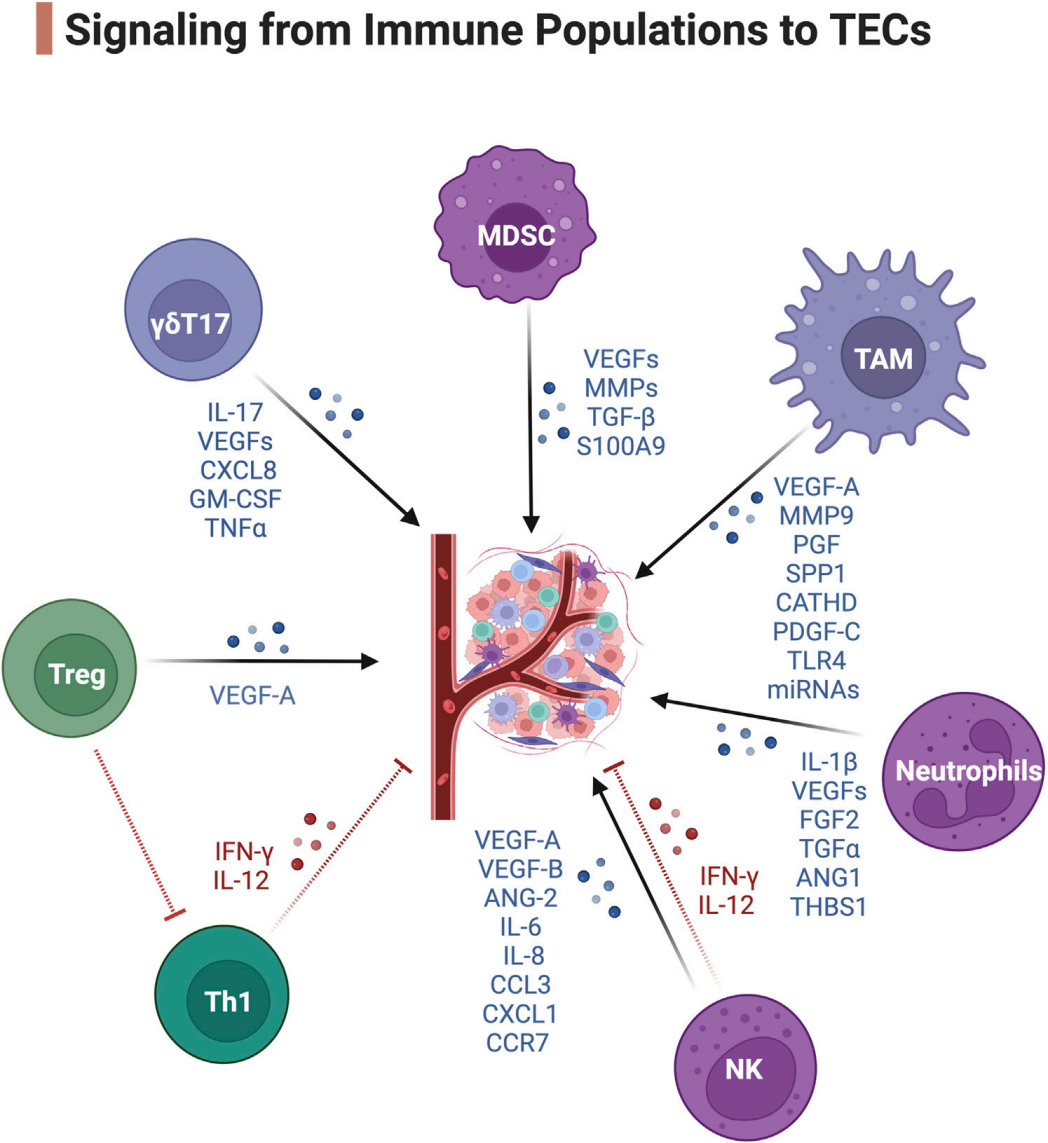
Signaling from immune populations to TECs. Blue, pro-angiogenic signaling. Red, anti-angiogenic signaling. Created with BioRender.com.

TECs also directly regulate immune cell survival and differentiation. Indoleamine-pyrrole 2,3-dioxygenase 1 (IDO1) is predominantly expressed in TECs. The depletion of L-arginine and L-tryptophan in tumors mediated by IDO1 directly inhibits proliferation and promotes apoptosis of CTLs (Mondanelli et al., 2017). The product of IDO1, l-kynurenine, activates the arylhydrocarbon receptor which promotes the differentiation of effector T cells into Tregs and upregulates IDO1 expression in DCs (Grohmann and Puccetti, 2015). Moreover, VEGF-A, IL-10 and PGE2 induce the death mediator Fas ligand (FasL) expression in TECs. FasL selectively induces apoptosis in CTLs but not Tregs because of high c-FLIP expression in Tregs (Motz et al., 2014). Galectin-1 (GAL1) reprograms the TECs to upregulate PD-L1 and death signal GAL9 and therefore drives T cell exclusion from the TME and is shown to exacerbate immunotherapy resistance in head and neck cancer (Nambiar et al., 2019). For NK cells, TECs upregulate RAE-1ε expression to internalize NKG2D that desensitizes the antitumor response of NK cells (Thompson et al., 2017). Recent data have shown that disarming of NK cells and monocytes happens rapidly and TECs may provide an explanation for this observation (Figure 2, created with BioRender.com).

## Signaling from immune cells to TECs

### TAMs

Perivascular TAMs are a highly specialized TAM subset that reside proximal, within 1 cell thickness, to the blood vasculature and can collaborate with MDSCs to form a multicellular pre-metastatic niche (Wang et al., 2017). These Tie2+ TAMs express angiogenic genes including VEGF-A, MMP9, PGF, SPP1, and CATHD (Movahedi et al., 2010) and have superior proangiogenic activity. The release of VEGF-A from these Tie2+ TAMs can induce endothelial tip cell formation and facilitate blood vessel anastomosis (the joining of two blood vessels) (Fantin et al., 2010). Moreover, perivascular TAMs often accumulate after irradiation (Kioi et al., 2010) and chemotherapy and stimulate transient vascular permeability via VEGF-A and promote tumor cell dissemination and tumor relapse (Harney et al., 2015). The secretion of the growth factor PDGF-C from perivascular TAMs also promotes pericyte expansion through PDGFRα on a population of pericyte-like mesenchymal cells (Opzoomer et al., 2021). TAMs can also activate TECs via tenascin C stimulation of Toll-like receptor four and trigger the formation of pro-metastatic vasculature and the dissemination of cancer cells (Hongu et al., 2022). M2d-phenotype TAMs also promote tumor-associated vessel growth by secreting miRNAs containing macrophage-derived exosomes (MDEs). It has been shown that these MDEs have an altered miRNA cargo profile (Binenbaum et al., 2018) in tumors that promote the proliferation of endothelial cells and increase tumor-associated vascular density. MDEs can transport miR-155-5p and miR-221-5p to induce angiogenesis in pancreatic ductal adenocarcinoma by inhibiting E2F2 in ECs (Yang et al., 2021).

### MDSCs

CD11b+Gr1+ MDSCs are another major source of soluble VEGFs promoting angiogenesis in tumors (Shojaei et al., 2009). MDSCs interact with TAMs to orchestrate the formation of dysfunctional TECs (Fang et al., 2023). Both TAMs and MDSCs can render tumors non-responsive to VEGF/VEGFR inhibition therapy (Rivera and Bergers, 2015) and lead to tumors reinitiating angiogenesis. Aside from VEGF-mediated angiogenesis, polymorphonuclear MDSCs express abundant amounts of MMP2, MMP8, MMP9, MMP13, and MMP14 (Binsfeld et al., 2016) to remodel the ECM. This process facilitates tumor intravasation as well as angiogenesis in collaboration with TAMs and NK cells (Binsfeld et al., 2016). MDSCs also produce TGF-β to induce tumor angiogenesis by activating fibroblasts to produce ECM adhesion molecules and stimulate blood vessel anastomosis (van Meeteren et al., 2011). S100 calcium-binding protein A9 (S100A9) secreted mainly via MDSCs can promote the angiogenesis of multiple myeloma (De Veirman et al., 2017). Importantly, these soluble factors are enriched in the exosomes derived from MDSCs, which is an important transporter to deliver pro-angiogenic signals to TECs (Zoller, 2018).

### Neutrophils

Tumor-associated neutrophils (TAN) can secrete proangiogenic or antiangiogenic factors. The hypoxic TME tunes the TANs to an anergic N2-phenotype in response to TGFβ (Fridlender et al., 2009) and induces them to secrete the pro-angiogenic factors IL-1β, VEGF-A, FGF2, TGFα and ANG1 (De Palma et al., 2017). N2-TANs lack expression of TIMP1 and as a consequence they express high level of free MMP9 as a catalytic angiogenesis driver and ECM remodeler in the TME (Ardi et al., 2007). On the contrary, TANs can release the antiangiogenic factor thrombospondin-1 regulated by peroxisome proliferator-activated receptor α (PPARα), preventing tumor growth (Kaipainen et al., 2007).

### NK cells

Although NK cells are primarily defined as a population of innate lymphoid cells with cytotoxic and cytokine-producing ability, their function in tumors expands well beyond their cytolytic potential. A specialized subset of decidual NK (dNK) cells has been discovered to promote vascularization during embryonic and placental development (Zhang et al., 2011). In tumors, the anergic dNK-like CD56hiCD16- NK cells with low degranulation capacity also develop pro-angiogenic phenotypes (Albini and Noonan, 2021). NKs are also another major source of soluble VEGFs (Gaggero et al., 2020), IL-8 and PLGF (Bruno et al., 2013). STAT3/STAT5 activation in tumor-infiltrating NKs also enhances the expression of ANG1/2, MMP-2, and tissue inhibitor of MMP (TIMP) in patients with colorectal cancer (Bruno et al., 2018). A study in renal cell carcinoma demonstrated this large group of CD56hiCD16- NK cells express an array of pro-angiogenic factors including VEGF-A, VEGF-B, ANG-2, IL-6, IL-8, CCL3, CXCL1, CCR7 and CD146 receptor (Guan et al., 2020). On the contrary, the cytotoxic NK cells together with CTLs produce IL-12 and IFN-γ that are key factors to suppress neovascularization in tumors (Yao et al., 1999). Yet the IFN-γ signaling has a controversial role in neovascularization. It has also been demonstrated that IFN-γ produced by NK cells and T cells during transmigration can escalate the angiogenesis potential by downregulating anti-neovascularization factor TNFSF15 expression in TECs (Lu et al., 2014).

### T cells

High Treg density has been associated with high intratumoral vessel density in renal cell carcinoma (Zhan et al., 2012) and endometrial adenocarcinoma (Giatromanolaki et al., 2008). Our recent study demonstrated preferential firm adhesion of Tregs onto TEC layers from clear cell renal cell carcinoma tumors compared with NECs (Xu et al., 2023). Recruited by hypoxia-induced chemokine CCL28 secreted by TECs, Tregs are reported to enhance VEGF-A levels in acute lymphoblastic leukemia (Li et al., 2018) and ovarian cancer (Facciabene et al., 2011). Tregs can also indirectly promote angiogenesis by modulating the function of other immune cells. Tregs inhibit activation of tumor-primed CD4 T cells, therefore suppressing IFN-γ-dependent antiangiogenic pathways (Casares et al., 2003). Of note, IFN-γ mainly secreted by Th1 CD4 T cells is known for inducing endothelial cell destruction (Beatty and Paterson, 2001). Moreover, it has been reported that the tumor-infiltrating γδT17 subset mediating pro-angiogenic IL-17 thereby inducing the expression of VEGF-A, CXCL8, GM-CSF, and TNFα release, can actively participate in the angiogenic process and the recruitment of MDSCs (Wu et al., 2014). IL-17 also indirectly promotes angiogenesis by activating the expression of CCL17 and CCL22 and facilitates Treg cell migration to tumor (Shibabaw et al., 2023).

## Conclusion and future perspectives

TECs have emerged as a signaling hub within the TME orchestrating a range of pro-tumoral functions. TECs have dual functions to diminish anti-tumor immune cell infiltration at the same time enhance immunosuppressive lymphocyte recruitment and metastatic tumor cell intravasation in the same tumor. Although many studies have been conducted to better understand TEC phenotypes, the spatial-temporal regulation of these dual functions remains an open research question. ECs are polarized cells with distinct molecular expression on the luminal and basal side regulating distinct biological processes. Microenvironmental cues such as hypoxia and nutrient gradients cues can lead to spatial heterogeneity in gene expression among TECs located in different regions of the tumor. The functional adaptation of TECs in subregions of solid tumors, and high-resolution dissection of signaling pathways on basal and lumen side of TECs remains to be elucidated. Moreover, the conventional concept of cell-cell interactions involves 2 cells: the signal receiver and deliverer. However, TECs can receive signals from different cell types such as immune cells, pericytes, tumor cells and stromal cells. Different cell types can enhance the signal in TECs through the same or synergistic molecules or attenuate the signal through counteracting signaling molecules. For example, VEGF-A is secreted by tumor cells, TECs, CAFs, MDSCs, TAMs, TANs and NKs, promoting neovascularization in tumor, while cytotoxic T cells and NK cells, although their entries are limited in tumor microenvironment, secrete the pro-inflammatory vessel activator IFN-γ to TECs. At the same time, immune cells secrete VEGFs, FGF2 to TECs to foster anergic ECs with dampened expression of ICAM-1 or VCAM-1. Throughout tumor progression and metastasis, TEC gene expression may be further altered by evolving tumor-stroma interactions, changes in tumor perfusion, and adaptation to therapeutic interventions. Studying only the paired interactions of two model cell types *in vitro* can greatly bias the conclusion. To date, few studies have analyzed the multi-cell type interactions surrounding TECs at a series of therapeutically critical time-points in solid tumors. Therefore, the relative signal intensities and outcomes in a spatial-temporal regulated manner need further exploration to facilitate the design of therapeutic agents targeting the key molecules that correct the overall phenotype of the TECs.

New technologies can empower future studies to better investigate these topics. Current single-cell sequencing and protein probing techniques are empowering studies to investigate the phenotypes and transcriptomic gene expression in all cell types with single-cell resolution in the TME to gain a complete picture of signaling from and toward TECs. Single-cell studies focused on TECs from different solid tumors have identified functional TEC subpopulations specific and common to certain types of tumors as well as the distinct adhesive interaction of TECs with immune cells within tumor (Sharma et al., 2020; Geldhof et al., 2022; Xu et al., 2024). Spatial genomics at a single-cell level, multiplex IHC, and high resolution *in situ* imaging can also now be deployed to examine the adhesion molecules and chemokine disposition on the luminal and basal side in relation with the presence of other cell types. Furthermore, vascularized microfluidic 3D chips can be used to model multi-cellular interactions using tumor spheroids to depict a road map of immune cell infiltration from TECs to tumor cells across the ECM (Miller et al., 2018; Miller et al., 2023). Future studies are expected to use these technologies to unravel the dynamics between TECs and immune responses in solid tumors, to discover and model therapeutical targets that involves multiple cell types.

In conclusion, TECs influence the function and recruitment of different immune cell populations shaping the TME. Yet the immune populations exert pro or antiangiogenic function inextricably linked to the TME with complex cell components. Research on the phenotype of TECs and the dissected interactions between TECs and immune components have depicted TECs as a signaling hub in promoting tumor pathogenesis. Meanwhile, the precisely regulated pro-angiogenic mechanisms by which immune cells exert to the TECs remain to be fully elucidated. Further investigations dissecting the spatial-temporal immunoregulatory function of TECs and the pro-angiogenic functions of TILs will be critical to establish the effective therapeutic targets that normalize pathologic neovascularization and counteract anergic immune functions in solid tumors.
